# Di-μ-acetato-κ^4^ *O*:*O*′-bis­[(1,10-phenanthroline-κ^2^ *N*,*N*′)(tri­fluoro­methane­sulfonato-κ*O*)copper(II)]

**DOI:** 10.1107/S1600536813027323

**Published:** 2013-10-12

**Authors:** Nanthawat Wannarit, Chaveng Pakawatchai, Sujittra Youngme

**Affiliations:** a Materials Chemistry Research Unit, Department of Chemistry and Center of Excellence for Innovation in Chemistry, Faculty of Science, Khon Kaen University, Khon Kaen 40002, Thailand; b Department of Chemistry, Faculty of Science, Prince of Songkla University, Hat Yai, Songkhla 90112, Thailand

## Abstract

The complete molecule of the title compound, [Cu_2_(C_2_H_3_O_2_)_2_(CF_3_O_3_S)_2_(C_12_H_8_N_2_)_2_], is completed by the application of a twofold rotation and comprises two Cu^II^ ions, each of which is penta­coordinated by two N atoms from a bidentate 1,10-phenanthroline (phen) ligand, two O atoms from acetate ligands and an O atom from a tri­fluoro­methane­sulfonate anion, forming a (4 + 1) distorted square-pyramidal coordination geometry. The Cu^II^ ions are connected by two acetate bridges in a *syn*–*syn* configuration. The F atoms of the tri­fluoro­methane­sulfonate ligands are disordered, with site-occupation factors of 70 and 30. The molecular structure is stabilized by intra­molecular face-to-face π–π inter­actions with centroid–centroid distances in the range 3.5654 (12)–3.8775(12) Å. The crystal structure is stabilized by C—H⋯O interactions, leading to a three-dimensional lattice structure.

## Related literature

For general background to this work, see: Moreira *et al.* (2007 ▶); Calvo *et al.* (2011 ▶); Reinoso *et al.* (2005 ▶, 2007 ▶); Ritchie *et al.* (2006 ▶); Wang *et al.* (2006 ▶). For literature used in the synthetic procedures, see: Youngme *et al.* (2008 ▶). For a related crystal structure, see: Tokii *et al.* (1990 ▶). For potential applications, see: Hill & Brown (1986 ▶); Mansuy *et al.* (1991 ▶); Hill & Zhang (1995 ▶). For an explanation of the τ parameter, see: Addison *et al.* (1984 ▶). For spectroscopic properties, see: Castro *et al.* (1992 ▶); Sletten & Julve (1999 ▶).
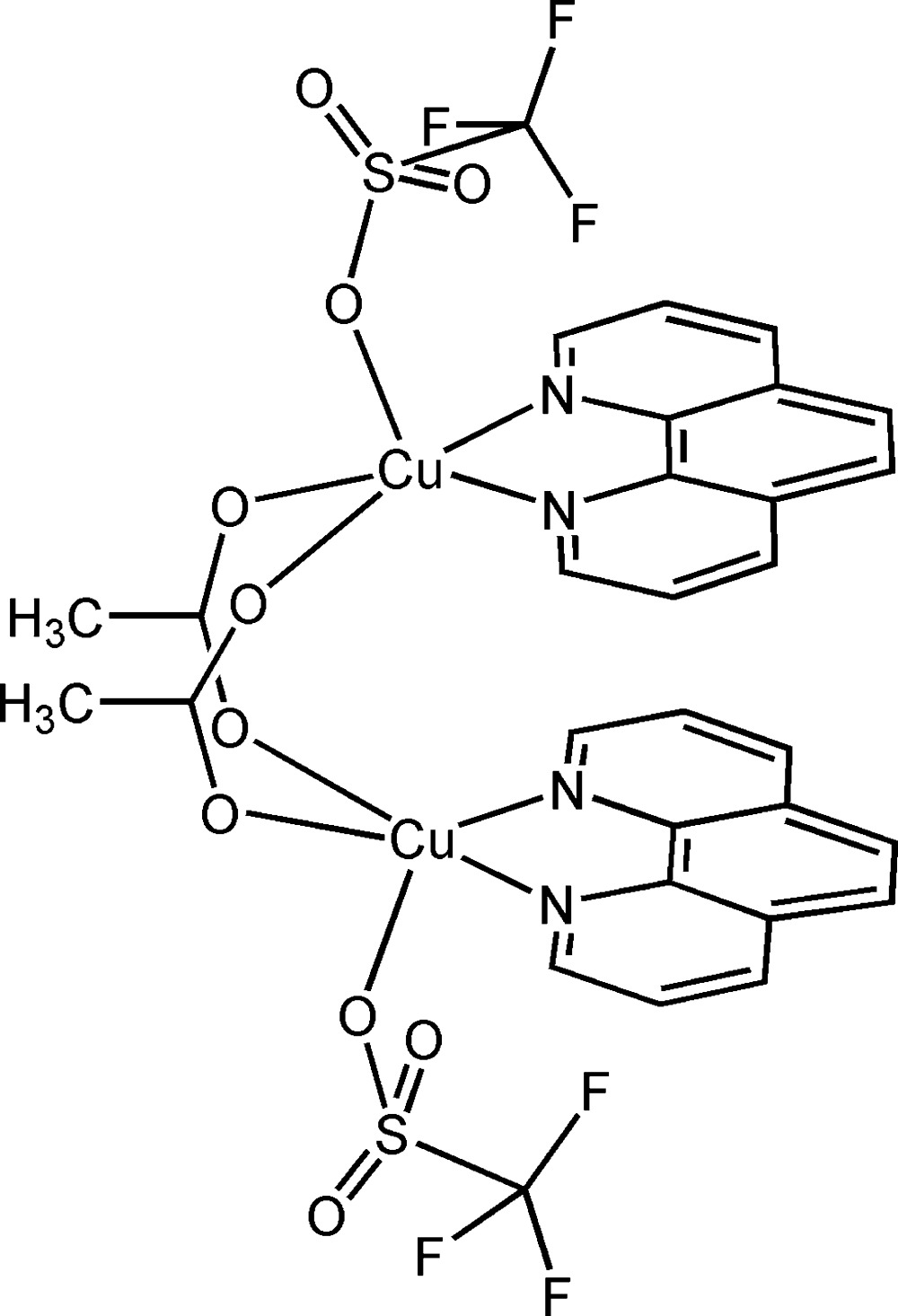


## Experimental

### 

#### Crystal data

[Cu_2_(C_2_H_3_O_2_)_2_(CF_3_O_3_S)_2_(C_12_H_8_N_2_)_2_]
*M* *_r_* = 903.72
Monoclinic, 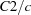

*a* = 13.1198 (5) Å
*b* = 16.1282 (6) Å
*c* = 16.3659 (6) Å
β = 95.507 (1)°
*V* = 3447.0 (2) Å^3^
*Z* = 4
Mo *K*α radiation
μ = 1.45 mm^−1^
*T* = 293 K
0.24 × 0.21 × 0.18 mm

#### Data collection

Bruker SMART APEX CCD diffractometer
Absorption correction: multi-scan (*SADABS*; Sheldrick, 2000 ▶) *T*_min_ = 0.872, *T*_max_ = 1.000
23313 measured reflections
4178 independent reflections
3491 reflections with *I* > 2σ(*I*)
*R*_int_ = 0.022

#### Refinement

*R*[*F* ^2^ > 2σ(*F* ^2^)] = 0.034
*wR*(*F* ^2^) = 0.102
*S* = 1.04
4178 reflections
272 parameters
H-atom parameters constrained
Δρ_max_ = 0.37 e Å^−3^
Δρ_min_ = −0.32 e Å^−3^

### 

Data collection: *SMART* (Bruker, 2000 ▶); cell refinement: *SMART*; data reduction: *SAINT* (Bruker, 2000 ▶) and *SHELXTL* (Sheldrick, 2008 ▶); program(s) used to solve structure: *SHELXTL*; program(s) used to refine structure: *SHELXL97* (Sheldrick, 2008 ▶); molecular graphics: *ORTEP-3 for Windows* (Farrugia, 2012 ▶) and *Mercury* (Macrae *et al.*, 2006 ▶); software used to prepare material for publication: *PLATON* (Spek, 2009 ▶) and *pubCIF* (Westrip, 2010 ▶).

## Supplementary Material

Crystal structure: contains datablock(s) global, I. DOI: 10.1107/S1600536813027323/im2439sup1.cif

Structure factors: contains datablock(s) I. DOI: 10.1107/S1600536813027323/im2439Isup2.hkl

Additional supplementary materials:  crystallographic information; 3D view; checkCIF report

## Figures and Tables

**Table 1:** Hydrogen-bond geometry (Å, °)

*D*—H⋯*A*	*D*—H	H⋯*A*	*D*⋯*A*	*D*—H⋯*A*
C1—H1⋯O5^i^	0.93	2.43	3.229 (3)	144
C3—H3⋯O4^ii^	0.93	2.34	3.213 (3)	157
C8—H8⋯O3^iii^	0.93	2.56	3.421 (3)	154

Symmetry codes: (i) 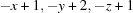; (ii) 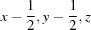; (iii) 
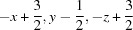
.

**Fig. 1.**
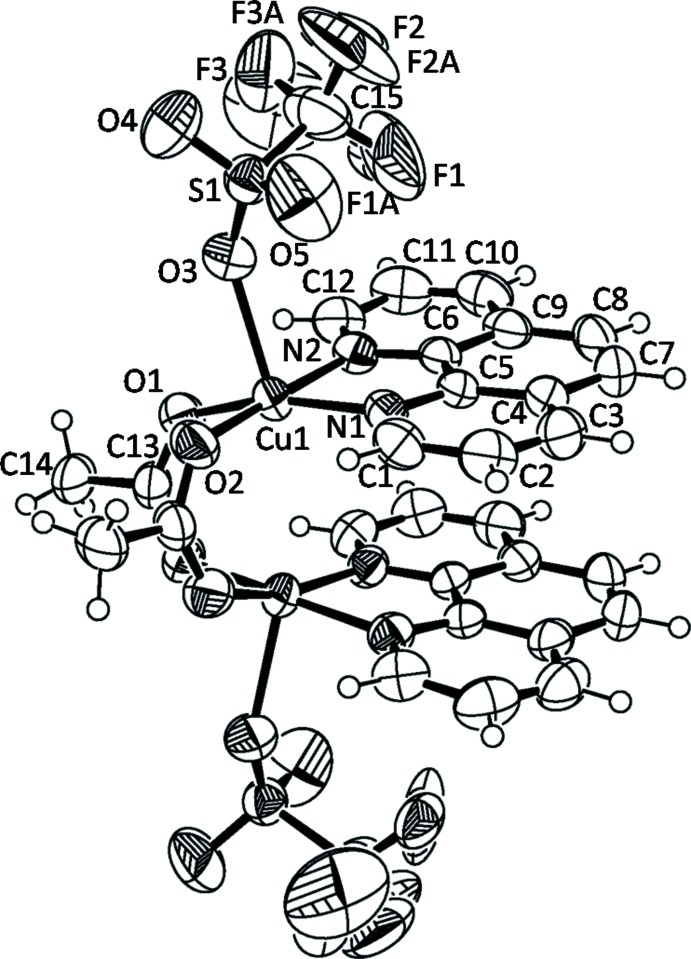
Molecular structure and atomic numbering scheme with thermal ellipsoids shown at 50% probability level.

**Fig. 2.**
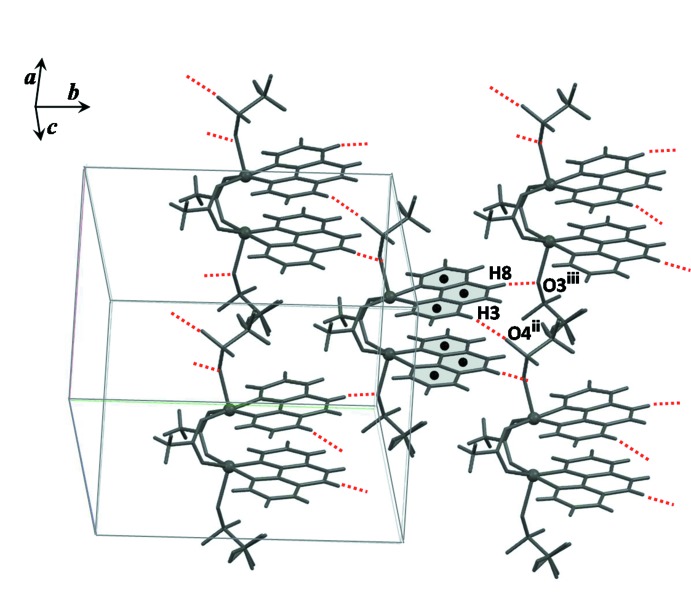
The crystal packing. View of two-dimensional layer constructed by intermolecular hydrogen bonding and view of the intramolecular face-to-face π-π interactions between aromatic rings of phen ligands.

**Fig. 3.**
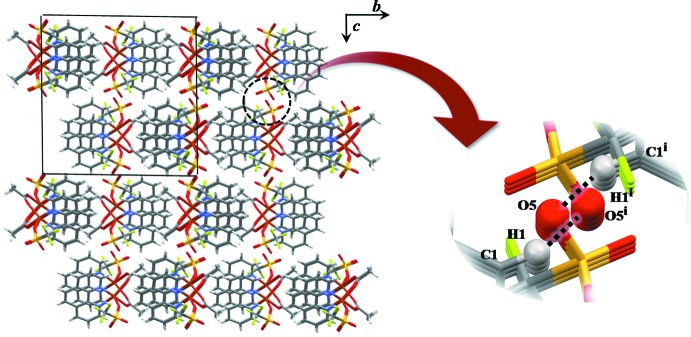
The crystal packing. View of three-dimensional framework (side view) constructued by intermolecular hydrogen bonding interactions between two-dimensional layers (C1—H1···O5i, symmetry code: (i) = -x+1, -y+2, -z+1).

## supplementary crystallographic information

### 1. Comment

The synthesis and characterization of polycarboxylato-bridged dinuclear copper(II) compounds namely dinuclear tetracarboxylato-bridged Cu^II^ compounds (paddlewheel-like structure) (*e.g.* Moreira *et al.*, 2007; Youngme *et al.*, 2008) and also dinuclear Cu^II^ compounds containing dicarboxylato-bridges (Tokii *et al.*, 1990; Reinoso *et al.*, 2005; Ritchie *et al.*, 2006) have attacted much attention in several years. These compounds have been prepared with the aim of studying their intramolecular magnetic properties which are determined predominantly by strong antiferromagnetic interactions. In addition, the dicarboxylato-bridged dinuclear Cu^II^ compounds have been frequently used as the models for the basic understanding of their magneto-structural correlations in theoretical studies (Moreira *et al.*, 2007; Calvo *et al.*, 2011). Copper(II) compounds containing doubly acetato-bridged dinuclear units, [Cu(phen)(µ-OOCCH_3_)_2_Cu(phen)]^2+^ (where phen = 1,10-phenanthroline), have also been shown to exhibit antiferromagnetic behavior (Tokii *et al.*, 1990). Furthermore, this type of dinuclear unit was used as the secondary building block in functionalized polyoxometalate (POMs) materials (Wang *et al.*, 2006; Reinoso *et al.*, 2007; Calvo *et al.*, 2011) to extend the dimensionality of structures leading to new hybrid materials and more selective applications, for example catalytic properties in organic oxidations (Hill & Brown, 1986; Mansuy *et al.*, 1991; Hill, & Zhang, 1995).

A new doubly acetato-bridged dinuclear Cu^II^ compound containing additional trifluoromethanesulfonate anions has been synthesized and its structural features are reported here. Compound I, bis((µ-acetato)(trifluoromethanesulfonato)(1,10-phenanthroline))dicopper(II) crystallized in the space group *C*2/*c* with an asymmetric unit containing one half of the dinuclear unit (Fig.1). This dinuclear unit has *C*_2_ symmetry around the *b* axis with Cu···Cu distance of 3.0309 (4) Å. Structurally, compound I consists of two [Cu(phen)(OSO_2_CF_3_)]^+^ cations connected together by two bridging acetato ligands in a *syn*-*syn* configuration. Both Cu^II^ atoms exhibit five coordination of CuN_2_O_2_O' chromophore, with the basal plane consisting of two phen N atoms [Cu—N = 2.0153 (18) and 1.9980 (18) Å] and two O atoms from acetate ligands [Cu—O = 1.9387 (17) and 1.9377 (18)]. Due to symmetry both square planes are parallel to one another. The apical position at Cu^II^ is occupied by an O atom from trifluoromethanesulfonate anion [Cu—O = 2.261 (2)], leading to the (4 + 1) square-pyramidal geometry. The square base of Cu^II^ chromophore is not perfectly planar, with the tetrahedral twist of 16.52 (7)° and Cu^II^ is situated above the basal plane by 0.14 (1) Å pointing towards the O atom of the trifluoromethanesulfonate anion. The distortion of a square pyramid can be best described by the structural parameter τ (τ = 0 for a square pyramid and τ =1 for a trigonal bipyramid (Addison *et al.*, 1984)), with τ = 0.23 for the title compound. The molecular structure of I reveals intramolecular face-to-face π-π interaction between aromatic rings of phen ligands (Fig. 1). Phenanthroline molecules are parallel with an average contact and angle of phen planes of 3.63 (3) Å and 5.96 (3)°, respectively. In general, the ligands are featureless: neither of phen group departs significantly from planarity [maximum deviations: 0.082 Å for C11 and 0.099 Å for C10 of *Cg*3 ring(N2, C6, C9, C10, C11, C12)] and the C—O bonds in the acetato bridging ligands display an almost perfect resonance [C13≐ O1 = 1.257 (3) Å and C13≐O2 = 1.250 (3) Å]. The crystal structure of compound I is determined by intermolecular hydrogen bonding interactions between methyl groups of acetato ligands (H14A) or phen ligands (H7 as hydrogen bond donor sites and H10) and oxygen/fluoride accepetors at trifluoromethanesulfonate anions (O3, O5 and F3) (see Table 1), generating two-dimensional layers parallel to the *ab* plane (Fig. 2). Moreover, these two-dimensional sheets are interconnected by hydrogen bond interactions between C—H of phen ligands and oxygen atoms of trifluoromethanesulfonate anions [C1—H1···O5^i^; symetry code (i) = -*x*+1, -*y*+2, -*z*+1] (see Table 2) in direction of crystallographic *c* axis, leading to three-dimensional lattice structure (Fig. 3). Although containing the same [(phen)Cu(µ-OOCCH_3_)_2_Cu(phen)]^2+^ unit, the structural topology of I is distinct from that of the related compound [Cu(phen)(µ-O_2_CCH_3_)(H_2_O)]_2_(NO_3_)_2_.4H_2_O (Tokii *et al.*, 1990) in which the apical position is occupied by water molecule. The dinuclear unit of this related compound also crystallized in *C*2/*c* space group and has *C*_2_ symmetry around the *b* axis with Cu···Cu distance of 3.063 Å, but its crystal lattice is mainly stabilized by intra- and intermolecular π-π interactions, generating a one-dimensional chain-like sructure. It is clear that the difference of the structural topology between compound I and the related compound caused by the effect of coordinated trifluoromethanesulfonate anions whereas nitrate anions are not coordinated to Cu in the other structure.

The diffuse reflectance spectrum of I displays a broad band at 15400 cm^-1^ and a lower energy shoulder at 14300 cm^-1^. This feature corresponds to a dominantly distorted square pyramidal geometry of Cu^II^ ions and is consistent with the observed structural parameters. The transitions may be assigned as d_xy_, d_yz_, d_xz_→ d_x2-y2_ and d_z2_→ d_x2-y2_. The IR spectrum of I, in addition to the phen vibrations shows the broad and intense bands of the stretching of the ionic CF_3_SO_3_^-^ at 1276 ν_as_(S–O), 1158 ν_as_(C–F) and 1031 ν_s_(S–O) cm^-1^ (Castro *et al.*, 1992). The IR spectrum also shows two broad and intense bands at 1567 and 1385 cm^-1^, corresponding to the ν_as_(COO^-^) and ν_s_(COO^-^) vibrations of acetate bridging ligands. The latter spectral properties completely disappear for related mononuclear compounds as [Cu(phen)_3_](CF_3_SO_3_)_2_.H_2_O (Sletten & Julve, 1999).

### 2. Experimental

A warm ethanolic solution (25 ml) of phen (0.198 g, 1.0 mmol) was added to a warm aqueous solution (15 ml) of Cu(CF_3_SO_3_)_2_ (0.370 g, 1.0 mmol). Then NaO_2_CCH_3_ solid (0.124 g, 1.0 mmol) was added to the mixture, yielding a clear dark blue solution. After a week, the blue rectangle-shaped crystals of compound I were obtained. The crystals were filtered off, washed with mother liquor and air-dried. Yield: *ca* 45%. Anal. Calc. for Cu_2_C_30_H_24_N_4_O_10_F_6_S_2_: C, 39.78; H, 2.67; N, 6.19%. Found: C, 39.12; H, 2.51; N, 6.36%.

### 3. Refinement

All H atoms were constrained to ideal positions, with C—H = 0.93 Å and *U*_iso_(H) =1.2U_eq_(C) for H atoms at phen and C—H = 0.96 Å and *U*_i__s__o_(H) =1.2U_eq_(C) for H atoms of acetate groups. Fluorine atoms of the trifluoromethanesulfonato ligands are disordered with site occupation factors of 70:30%.

### Crystal data

**Table:**

[Cu_2_(C_2_H_3_O_2_)_2_(CF_3_O_3_S)_2_(C_12_H_8_N_2_)_2_]	*F*(000) = 1816
*M**_r_* = 903.72	*D*_x_ = 1.741 Mg m^−^^3^
Monoclinic, *C*2/*c*	Mo *K*α radiation, λ = 0.71073 Å
Hall symbol: -C 2yc	Cell parameters from 8485 reflections
*a* = 13.1198 (5) Å	θ = 2.3–26.4°
*b* = 16.1282 (6) Å	µ = 1.45 mm^−^^1^
*c* = 16.3659 (6) Å	*T* = 293 K
β = 95.507 (1)°	Block, blue
*V* = 3447.0 (2) Å^3^	0.24 × 0.21 × 0.18 mm
*Z* = 4	

### Data collection

**Table:**

Bruker SMART APEX CCD diffractometer	4178 independent reflections
Radiation source: fine-focus sealed tube	3491 reflections with *I* > 2σ(*I*)
Graphite monochromator	*R*_int_ = 0.022
phi and ω scans	θ_max_ = 28.1°, θ_min_ = 2.0°
Absorption correction: multi-scan (*SADABS*; Sheldrick, 2000)	*h* = −17→17
*T*_min_ = 0.872, *T*_max_ = 1.000	*k* = −21→21
23313 measured reflections	*l* = −21→21

### Refinement

**Table:**

Refinement on *F*^2^	Primary atom site location: structure-invariant direct methods
Least-squares matrix: full	Secondary atom site location: difference Fourier map
*R*[*F*^2^ > 2σ(*F*^2^)] = 0.034	Hydrogen site location: inferred from neighbouring sites
*wR*(*F*^2^) = 0.102	H-atom parameters constrained
*S* = 1.04	*w* = 1/[σ^2^(*F*_o_^2^) + (0.0624*P*)^2^ + 1.5014*P*] where *P* = (*F*_o_^2^ + 2*F*_c_^2^)/3
4178 reflections	(Δ/σ)_max_ = 0.001
272 parameters	Δρ_max_ = 0.37 e Å^−^^3^
0 restraints	Δρ_min_ = −0.32 e Å^−^^3^

### Special details

**Table:**

Geometry. Bond distances, angles *etc*. have been calculated using the rounded fractional coordinates. All e.s.d.'s are estimated from the variances of the (full) variance-covariance matrix. The cell e.s.d.'s are taken into account in the estimation of distances, angles and torsion angles

### Fractional atomic coordinates and isotropic or equivalent isotropic displacement parameters (Å^2^)

**Table:**

	*x*	*y*	*z*	*U*_iso_*/*U*_eq_	Occ. (<1)
Cu1	0.59608 (2)	0.99743 (1)	0.70547 (2)	0.0458 (1)	
S1	0.78035 (4)	1.01647 (3)	0.56674 (3)	0.0539 (2)	
F1	0.8335 (6)	0.8733 (4)	0.6161 (4)	0.176 (4)	0.700
F2	0.9340 (5)	0.9174 (4)	0.5429 (4)	0.137 (2)	0.700
F3	0.9359 (6)	0.9687 (6)	0.6618 (6)	0.199 (4)	0.700
O1	0.63242 (11)	1.07473 (9)	0.79413 (10)	0.0626 (5)	
O2	0.51246 (12)	1.08054 (9)	0.64547 (10)	0.0608 (5)	
O3	0.73435 (13)	1.03226 (11)	0.64052 (11)	0.0712 (6)	
O4	0.8331 (2)	1.08491 (14)	0.53822 (17)	0.1123 (10)	
O5	0.7149 (2)	0.97542 (18)	0.50650 (17)	0.1224 (11)	
N1	0.53445 (12)	0.90229 (9)	0.63975 (10)	0.0449 (5)	
N2	0.68014 (12)	0.90588 (10)	0.76211 (9)	0.0472 (5)	
C1	0.46043 (16)	0.90316 (14)	0.57797 (12)	0.0556 (7)	
C2	0.41990 (18)	0.83081 (17)	0.54255 (14)	0.0640 (8)	
C3	0.45639 (18)	0.75590 (15)	0.56951 (15)	0.0633 (8)	
C4	0.53672 (15)	0.75244 (12)	0.63350 (13)	0.0509 (6)	
C5	0.57234 (13)	0.82808 (11)	0.66659 (11)	0.0423 (5)	
C6	0.65295 (13)	0.82997 (11)	0.73215 (11)	0.0430 (5)	
C7	0.58190 (18)	0.67773 (13)	0.66651 (17)	0.0649 (8)	
C8	0.65946 (18)	0.67936 (13)	0.72685 (16)	0.0642 (8)	
C9	0.69878 (15)	0.75606 (13)	0.76071 (13)	0.0526 (6)	
C10	0.78116 (18)	0.76280 (16)	0.82166 (15)	0.0664 (8)	
C11	0.81020 (18)	0.83952 (18)	0.85040 (15)	0.0701 (8)	
C12	0.75733 (17)	0.91011 (15)	0.82085 (13)	0.0605 (7)	
C13	0.57629 (16)	1.10508 (11)	0.84421 (13)	0.0514 (6)	
C14	0.6188 (2)	1.17618 (14)	0.89565 (17)	0.0706 (8)	
C15	0.8783 (3)	0.9414 (2)	0.5977 (2)	0.0953 (14)	
F3A	0.9596 (9)	0.9817 (7)	0.6337 (13)	0.153 (7)	0.300
F1A	0.8515 (8)	0.8809 (8)	0.6463 (7)	0.083 (3)	0.300
F2A	0.9048 (15)	0.9173 (13)	0.5189 (12)	0.193 (8)	0.300
H1	0.43520	0.95390	0.55800	0.0670*	
H2	0.36750	0.83360	0.50010	0.0770*	
H3	0.42880	0.70730	0.54610	0.0760*	
H7	0.55740	0.62700	0.64600	0.0780*	
H12	0.77660	0.96160	0.84290	0.0730*	
H14A	0.68970	1.18380	0.88760	0.1060*	
H14B	0.61240	1.16460	0.95250	0.1060*	
H14C	0.58140	1.22570	0.87990	0.1060*	
H8	0.68800	0.62970	0.74680	0.0770*	
H10	0.81550	0.71570	0.84220	0.0800*	
H11	0.86560	0.84480	0.89000	0.0840*	

### Atomic displacement parameters (Å^2^)

**Table:**

	*U*^11^	*U*^22^	*U*^33^	*U*^12^	*U*^13^	*U*^23^
Cu1	0.0501 (2)	0.0364 (1)	0.0522 (2)	0.0032 (1)	0.0111 (1)	−0.0044 (1)
S1	0.0583 (3)	0.0503 (3)	0.0537 (3)	0.0031 (2)	0.0083 (2)	0.0036 (2)
F1	0.280 (8)	0.069 (3)	0.194 (7)	0.081 (4)	0.099 (6)	0.051 (4)
F2	0.115 (3)	0.157 (5)	0.152 (4)	0.072 (3)	0.077 (4)	0.039 (4)
F3	0.128 (6)	0.280 (9)	0.172 (5)	0.051 (5)	−0.073 (5)	0.031 (5)
O1	0.0620 (9)	0.0553 (8)	0.0732 (10)	−0.0075 (7)	0.0200 (7)	−0.0240 (7)
O2	0.0659 (9)	0.0482 (7)	0.0716 (9)	0.0163 (7)	0.0235 (7)	0.0118 (7)
O3	0.0704 (10)	0.0658 (10)	0.0820 (11)	0.0007 (8)	0.0305 (9)	−0.0070 (9)
O4	0.135 (2)	0.0787 (14)	0.1311 (19)	−0.0137 (13)	0.0530 (16)	0.0345 (13)
O5	0.127 (2)	0.131 (2)	0.0992 (18)	0.0116 (17)	−0.0404 (16)	−0.0369 (16)
N1	0.0464 (8)	0.0425 (8)	0.0464 (8)	0.0047 (6)	0.0076 (6)	−0.0021 (6)
N2	0.0475 (8)	0.0505 (9)	0.0444 (8)	0.0024 (7)	0.0084 (6)	−0.0017 (6)
C1	0.0570 (11)	0.0594 (12)	0.0500 (11)	0.0093 (9)	0.0027 (9)	−0.0019 (9)
C2	0.0589 (12)	0.0782 (16)	0.0539 (12)	−0.0017 (10)	−0.0005 (9)	−0.0116 (10)
C3	0.0647 (13)	0.0629 (13)	0.0634 (13)	−0.0117 (10)	0.0125 (10)	−0.0195 (10)
C4	0.0527 (10)	0.0442 (9)	0.0586 (11)	−0.0023 (8)	0.0194 (9)	−0.0061 (8)
C5	0.0433 (8)	0.0400 (8)	0.0458 (9)	0.0023 (7)	0.0154 (7)	−0.0022 (7)
C6	0.0423 (8)	0.0433 (9)	0.0456 (9)	0.0042 (7)	0.0154 (7)	0.0017 (7)
C7	0.0725 (14)	0.0384 (10)	0.0869 (16)	−0.0017 (9)	0.0240 (13)	−0.0038 (10)
C8	0.0719 (14)	0.0399 (10)	0.0845 (16)	0.0131 (9)	0.0271 (12)	0.0100 (10)
C9	0.0505 (10)	0.0547 (11)	0.0554 (11)	0.0112 (8)	0.0189 (9)	0.0101 (9)
C10	0.0628 (13)	0.0766 (15)	0.0608 (13)	0.0211 (11)	0.0104 (10)	0.0149 (11)
C11	0.0553 (12)	0.1009 (19)	0.0528 (12)	0.0110 (12)	−0.0016 (10)	0.0062 (12)
C12	0.0567 (11)	0.0728 (14)	0.0515 (11)	−0.0018 (10)	0.0028 (9)	−0.0072 (10)
C13	0.0623 (11)	0.0364 (8)	0.0575 (11)	−0.0067 (8)	0.0154 (9)	−0.0053 (8)
C14	0.0791 (15)	0.0531 (12)	0.0838 (16)	−0.0205 (11)	0.0291 (13)	−0.0256 (11)
C15	0.083 (2)	0.098 (2)	0.109 (3)	0.0357 (18)	0.0305 (19)	0.0181 (19)
F3A	0.046 (3)	0.082 (5)	0.32 (2)	0.000 (3)	−0.039 (7)	0.004 (8)
F1A	0.093 (4)	0.075 (5)	0.087 (5)	0.038 (3)	0.042 (3)	0.038 (4)
F2A	0.165 (14)	0.234 (16)	0.185 (14)	0.086 (10)	0.037 (10)	−0.120 (12)

### Geometric parameters (Å, º)

**Table:**

Cu1—O1	1.9376 (16)	C3—C4	1.414 (3)
Cu1—O2	1.9385 (16)	C4—C5	1.397 (3)
Cu1—O3	2.2597 (18)	C4—C7	1.426 (3)
Cu1—N1	1.9995 (15)	C5—C6	1.433 (2)
Cu1—N2	2.0148 (16)	C6—C9	1.395 (3)
S1—O3	1.4237 (18)	C7—C8	1.349 (4)
S1—O4	1.406 (2)	C8—C9	1.431 (3)
S1—O5	1.409 (3)	C9—C10	1.403 (3)
S1—C15	1.803 (4)	C10—C11	1.365 (4)
F1—C15	1.295 (8)	C11—C12	1.395 (4)
F1A—C15	1.327 (13)	C13—C14	1.498 (3)
F2—C15	1.270 (7)	C1—H1	0.9302
F2A—C15	1.42 (2)	C2—H2	0.9304
F3—C15	1.309 (10)	C3—H3	0.9304
F3A—C15	1.337 (15)	C7—H7	0.9299
O1—C13	1.253 (3)	C8—H8	0.9300
O2—C13^i^	1.256 (3)	C10—H10	0.9295
N1—C1	1.333 (3)	C11—H11	0.9301
N1—C5	1.353 (2)	C12—H12	0.9307
N2—C12	1.329 (3)	C14—H14A	0.9598
N2—C6	1.354 (2)	C14—H14B	0.9605
C1—C2	1.386 (3)	C14—H14C	0.9596
C2—C3	1.358 (4)		
			
Cu1···O5	3.760 (3)	O4···H3^iv^	2.3363
Cu1···O1^i^	3.2474 (15)	O4···H11^v^	2.7477
Cu1···O2^i^	3.2315 (16)	O5···H14B^v^	2.7315
Cu1···N1^i^	3.5407 (16)	O5···H1^vi^	2.4275
Cu1···N2^i^	3.9955 (16)	N1···Cu1^i^	3.5407 (16)
Cu1···C1^i^	3.991 (2)	N2···F1A	3.103 (11)
Cu1···C5^i^	4.1970 (18)	N2···Cu1^i^	3.9955 (16)
Cu1···H8^ii^	3.5723	N2···C1^i^	3.344 (3)
F1···O3	2.920 (7)	C1···C12^i^	3.439 (3)
F1···O5	2.796 (7)	C1···O5^vi^	3.229 (3)
F1···C6	3.251 (8)	C1···N2^i^	3.344 (3)
F1···C14^iii^	3.249 (7)	C1···C13^i^	3.546 (3)
F1A···N2	3.103 (11)	C1···Cu1^i^	3.991 (2)
F1A···C6	3.184 (11)	C3···O4^vii^	3.213 (3)
F1A···C12	3.254 (11)	C3···C9^i^	3.599 (3)
F1A···O5	3.160 (12)	C5···C6^i^	3.525 (2)
F1A···O3	2.881 (12)	C5···Cu1^i^	4.1970 (18)
F2···O5	3.027 (7)	C5···C5^i^	3.472 (2)
F2···O4	3.006 (7)	C6···C5^i^	3.525 (2)
F2A···O4	2.89 (2)	C6···F1A	3.184 (11)
F2A···O5	2.65 (2)	C6···F1	3.251 (8)
F3···O4	2.983 (10)	C8···O1^iii^	3.256 (3)
F3···O3	2.826 (8)	C9···C3^i^	3.599 (3)
F3A···O4	2.733 (16)	C11···O4^viii^	3.293 (4)
F3A···O3	3.078 (12)	C12···C1^i^	3.439 (3)
F1···H14C^iii^	2.6275	C12···F1A	3.254 (11)
F1A···H14C^iii^	2.7013	C13···C13^i^	3.512 (3)
F3A···H7^iv^	2.6700	C13···C1^i^	3.546 (3)
O1···C8^ii^	3.256 (3)	C13···O5^viii^	3.335 (3)
O1···Cu1^i^	3.2474 (15)	C14···O5^viii^	3.228 (4)
O2···Cu1^i^	3.2315 (16)	C14···F1^ii^	3.249 (7)
O3···F3	2.826 (8)	C2···H14B^v^	3.0447
O3···F1	2.920 (7)	C8···H14A^iii^	2.8533
O3···F3A	3.078 (12)	C13···H1^i^	2.9284
O3···F1A	2.881 (12)	H1···C13^i^	2.9284
O4···F3	2.983 (10)	H1···O5^vi^	2.4275
O4···C3^iv^	3.213 (3)	H1···O2	2.6401
O4···F2	3.006 (7)	H3···H7	2.5822
O4···C11^v^	3.293 (4)	H3···O4^vii^	2.3363
O4···F2A	2.89 (2)	H7···F3A^vii^	2.6700
O4···F3A	2.733 (16)	H7···H3	2.5822
O5···Cu1	3.760 (3)	H8···H10	2.5804
O5···F2A	2.65 (2)	H8···Cu1^iii^	3.5723
O5···C13^v^	3.335 (3)	H8···O1^iii^	2.6624
O5···C14^v^	3.228 (4)	H8···O3^iii^	2.5589
O5···F2	3.027 (7)	H10···H8	2.5804
O5···F1A	3.160 (12)	H11···O4^viii^	2.7477
O5···F1	2.796 (7)	H12···O1	2.6934
O5···C1^vi^	3.229 (3)	H14A···C8^ii^	2.8533
O1···H12	2.6934	H14B···C2^viii^	3.0447
O1···H8^ii^	2.6624	H14B···O5^viii^	2.7315
O2···H1	2.6401	H14C···F1^ii^	2.6275
O3···H8^ii^	2.5589	H14C···F1A^ii^	2.7013
			
O1—Cu1—O2	91.21 (6)	C8—C9—C10	124.5 (2)
O1—Cu1—O3	92.37 (6)	C9—C10—C11	119.1 (2)
O1—Cu1—N1	162.62 (7)	C10—C11—C12	120.5 (2)
O1—Cu1—N2	92.44 (6)	N2—C12—C11	121.8 (2)
O2—Cu1—O3	91.76 (7)	O1—C13—C14	117.14 (19)
O2—Cu1—N1	94.48 (6)	O1—C13—O2^i^	125.13 (18)
O2—Cu1—N2	176.35 (6)	O2^i^—C13—C14	117.73 (19)
O3—Cu1—N1	103.84 (7)	S1—C15—F1	107.9 (4)
O3—Cu1—N2	88.28 (6)	S1—C15—F2	116.7 (4)
N1—Cu1—N2	81.97 (6)	S1—C15—F3	109.9 (5)
O3—S1—O4	113.79 (13)	S1—C15—F1A	116.1 (5)
O3—S1—O5	113.45 (13)	S1—C15—F2A	99.1 (8)
O3—S1—C15	103.33 (13)	S1—C15—F3A	108.3 (5)
O4—S1—O5	115.02 (16)	F1—C15—F2	102.1 (5)
O4—S1—C15	105.09 (16)	F1—C15—F3	109.8 (6)
O5—S1—C15	104.55 (16)	F2—C15—F3	110.0 (6)
Cu1—O1—C13	128.57 (14)	F1A—C15—F2A	116.4 (10)
Cu1—O2—C13^i^	129.53 (14)	F1A—C15—F3A	109.8 (9)
Cu1—O3—S1	141.19 (11)	F2A—C15—F3A	106.2 (12)
Cu1—N1—C1	128.86 (14)	N1—C1—H1	118.98
Cu1—N1—C5	112.78 (12)	C2—C1—H1	118.97
C1—N1—C5	118.28 (16)	C1—C2—H2	119.86
Cu1—N2—C6	112.49 (12)	C3—C2—H2	119.87
Cu1—N2—C12	129.82 (15)	C2—C3—H3	120.32
C6—N2—C12	117.66 (17)	C4—C3—H3	120.31
N1—C1—C2	122.0 (2)	C4—C7—H7	119.34
C1—C2—C3	120.3 (2)	C8—C7—H7	119.47
C2—C3—C4	119.4 (2)	C7—C8—H8	119.37
C3—C4—C5	116.78 (18)	C9—C8—H8	119.37
C3—C4—C7	124.6 (2)	C9—C10—H10	120.49
C5—C4—C7	118.66 (19)	C11—C10—H10	120.44
N1—C5—C4	123.22 (17)	C10—C11—H11	119.78
N1—C5—C6	116.49 (16)	C12—C11—H11	119.74
C4—C5—C6	120.29 (17)	N2—C12—H12	119.07
N2—C6—C5	116.13 (16)	C11—C12—H12	119.09
N2—C6—C9	124.13 (17)	C13—C14—H14A	109.53
C5—C6—C9	119.74 (17)	C13—C14—H14B	109.45
C4—C7—C8	121.2 (2)	C13—C14—H14C	109.45
C7—C8—C9	121.3 (2)	H14A—C14—H14B	109.50
C6—C9—C8	118.75 (19)	H14A—C14—H14C	109.50
C6—C9—C10	116.73 (19)	H14B—C14—H14C	109.39
			
O2—Cu1—O1—C13	68.10 (18)	Cu1—N1—C1—C2	−174.67 (16)
O3—Cu1—O1—C13	159.91 (17)	C5—N1—C1—C2	1.8 (3)
N2—Cu1—O1—C13	−111.71 (17)	Cu1—N1—C5—C4	176.12 (15)
O1—Cu1—O2—C13^i^	−78.62 (18)	Cu1—N1—C5—C6	−4.0 (2)
O3—Cu1—O2—C13^i^	−171.03 (18)	C1—N1—C5—C4	−0.9 (3)
N1—Cu1—O2—C13^i^	84.94 (18)	C1—N1—C5—C6	178.99 (17)
O1—Cu1—O3—S1	−175.37 (18)	Cu1—N2—C6—C5	0.7 (2)
O2—Cu1—O3—S1	−84.09 (18)	Cu1—N2—C6—C9	−179.80 (15)
N1—Cu1—O3—S1	10.96 (19)	C12—N2—C6—C5	−177.49 (17)
N2—Cu1—O3—S1	92.26 (18)	C12—N2—C6—C9	2.0 (3)
O2—Cu1—N1—C1	−0.85 (18)	Cu1—N2—C12—C11	−176.94 (16)
O2—Cu1—N1—C5	−177.49 (13)	C6—N2—C12—C11	0.9 (3)
O3—Cu1—N1—C1	−93.78 (17)	N1—C1—C2—C3	−1.1 (3)
O3—Cu1—N1—C5	89.58 (13)	C1—C2—C3—C4	−0.6 (3)
N2—Cu1—N1—C1	−180.00 (18)	C2—C3—C4—C5	1.4 (3)
N2—Cu1—N1—C5	3.37 (13)	C2—C3—C4—C7	−179.5 (2)
O1—Cu1—N2—C6	161.25 (13)	C3—C4—C7—C8	179.0 (2)
O1—Cu1—N2—C12	−20.80 (18)	C5—C4—C7—C8	−1.9 (3)
O3—Cu1—N2—C6	−106.45 (13)	C7—C4—C5—C6	0.2 (3)
O3—Cu1—N2—C12	71.50 (18)	C3—C4—C5—N1	−0.7 (3)
N1—Cu1—N2—C6	−2.21 (12)	C3—C4—C5—C6	179.43 (18)
N1—Cu1—N2—C12	175.74 (18)	C7—C4—C5—N1	−179.86 (19)
O4—S1—O3—Cu1	146.81 (18)	C4—C5—C6—C9	2.6 (3)
O5—S1—O3—Cu1	12.8 (2)	N1—C5—C6—N2	2.2 (2)
C15—S1—O3—Cu1	−99.8 (2)	N1—C5—C6—C9	−177.30 (17)
O3—S1—C15—F1	67.4 (4)	C4—C5—C6—N2	−177.90 (17)
O3—S1—C15—F2	−178.4 (4)	N2—C6—C9—C10	−3.1 (3)
O3—S1—C15—F3	−52.3 (5)	C5—C6—C9—C8	−3.8 (3)
O4—S1—C15—F1	−173.0 (4)	C5—C6—C9—C10	176.37 (18)
O4—S1—C15—F2	−58.8 (4)	N2—C6—C9—C8	176.76 (19)
O4—S1—C15—F3	67.2 (5)	C4—C7—C8—C9	0.6 (4)
O5—S1—C15—F1	−51.5 (4)	C7—C8—C9—C6	2.2 (3)
O5—S1—C15—F2	62.7 (4)	C7—C8—C9—C10	−178.0 (2)
O5—S1—C15—F3	−171.3 (5)	C8—C9—C10—C11	−178.5 (2)
Cu1—O1—C13—C14	−167.96 (15)	C6—C9—C10—C11	1.3 (3)
Cu1—O1—C13—O2^i^	12.1 (3)	C9—C10—C11—C12	1.4 (4)
Cu1—O2—C13^i^—O1^i^	−5.3 (3)	C10—C11—C12—N2	−2.6 (4)
Cu1—O2—C13^i^—C14^i^	174.68 (15)		

Symmetry codes: (i) −*x*+1, *y*, −*z*+3/2; (ii) −*x*+3/2, *y*+1/2, −*z*+3/2; (iii) −*x*+3/2, *y*−1/2, −*z*+3/2; (iv) *x*+1/2, *y*+1/2, *z*; (v) *x*, −*y*+2, *z*−1/2; (vi) −*x*+1, −*y*+2, −*z*+1; (vii) *x*−1/2, *y*−1/2, *z*; (viii) *x*, −*y*+2, *z*+1/2.

### Hydrogen-bond geometry (Å, º)

**Table:**

*D*—H···*A*	*D*—H	H···*A*	*D*···*A*	*D*—H···*A*
C1—H1···O5^vi^	0.93	2.43	3.229 (3)	144
C3—H3···O4^vii^	0.93	2.34	3.213 (3)	157
C8—H8···O3^iii^	0.93	2.56	3.421 (3)	154

Symmetry codes: (iii) −*x*+3/2, *y*−1/2, −*z*+3/2; (vi) −*x*+1, −*y*+2, −*z*+1; (vii) *x*−1/2, *y*−1/2, *z*.
